# The Pharmacopœia in the United States of America

**Published:** 1852-01

**Authors:** 


### ARTICLE V.

*Tne Pharmacopœia of the United Stutes of America.* By authority of the National Medical Convention held at Washington, A. D. 1850. Philadelphia: Lippincott, Grambo & Co., 1851.

We have already published a list of most of the new preparations that have been introduced into this edition of the work. The style in which it is published is remarkably good, its classification of preparations is plain, and its language is remarkably concise.

